# Widely Targeted Metabolomics Reveals the Quality Characteristics of a New Tea Cultivar, ‘Baiyun 0492’

**DOI:** 10.3390/foods14132206

**Published:** 2025-06-23

**Authors:** Ying Yu, Zijun Liang, Lei Zhang, Zhizhi Chen, Yixuan Zhao, Qiang Chen, Naixing Ye, Ruxing Yang

**Affiliations:** 1 Tea Research Institute, Fujian Academy of Agricultural Sciences, Fuzhou 350013, China; yuyingrose007@163.com (Y.Y.); sindy_0323@163.com (Z.L.); 18759188208@163.com (L.Z.); czz9999@126.com (Z.C.); z995811994@163.com (Y.Z.); m13193868172@163.com (Q.C.); 2 Fujian Branch of National Center for Tea Improvement, Fuzhou 350013, China; 3 College of Horticulture, Fujian Agriculture and Forestry University, Fuzhou 350002, China

**Keywords:** new tea tree stain, taste characteristics, flavor characteristics, metabolomics

## Abstract

To explore the taste characteristics and flavor characteristics of Baiyun 0492 white tea, Fuyun 6 was used as a comparison to describe and analyze the taste of the tea by the standard tea sensory evaluation method and quantitative descriptive analysis. A total of 1083 nonvolatile metabolites were identified by ultrahigh-performance liquid chromatography and mass spectrometry (UPLC–MS–MS). N-Feruloyl tyramine, with strong biological activity, may be the specific metabolite of Baiyun 0492 white tea, and 3,4,5-tricaffeoylquinic acid, with a bitter taste, may be the specific metabolite of Fuyun 6 white tea. Most of the different metabolite contents were negatively correlated with the Baiyun 0492 and Fuyun 6 white tea grades. KEGG metabolic pathway analysis showed that Baiyun 0492 and Fuyun 6 white teas had higher enrichment levels of flavonoids, caffeine, amino acids and carbohydrate-related metabolic pathways. Correlation analysis using widely targeted metabolomics and quantitative taste description showed that most flavonoid differences and some amino acid and phenolic acid differences were significantly positively correlated with bitterness and astringency, including quercetin-3-o-rhamnoside (quercetin), L-arginine, 3,4,5-tricaffioylquinic acid, and neochlorogenic acid. Some soluble sugars were positively correlated with sweetness, including D-maltose and D-sucrose, which were the key taste components of Baiyun 0492 and Fuyun 6. These metabolites may be responsible for the taste characteristics of Baiyun 0492, which is characterized as sweet and mellow, while the taste of Fuyun 6 is mainly mellow. In this study, a wide range of targeted metabolomics techniques were used to screen out different metabolites related to the quality of Baiyun 0492 and Fuyun 6 white teas, providing a reference for clarifying the quality characteristics of Baiyun 0492.

## 1. Introduction

White tea, one of six kinds of traditional tea in China, is mainly produced in the northeast of Fujian Province. Based on the different tenderness levels of fresh tea leaves, white tea can generally be classified into Baihaoyinzhen (bud only), Baimudan (one or two leaves with buds), Gongmei (more than two leaves with buds) and Shoumei (more than two leaves without buds) [1]. In recent years, white tea has received increasing attention worldwide, owing to its desirable flavors and various health benefits, which has led to studies of suitable cultivars of white tea and innovation in floral white tea products using highly aromatic cultivars [2]. The types, contents and proportions of nonvolatile compounds, including flavonoids, amino acids, phenolic acids and their derivatives, alkaloids, lipids, sugars and pigment substances in white tea directly affect the tea color, aroma, taste and other sensory qualities. Theanine, aspartic acid, asparagine and adenosine monophosphate are positively correlated with the umami taste of white tea, and flavan-3-ols (EGCG, ECG, GCG, and epigallocatechin 3-coumaroate), theasinensins (asinensin A and theasinensin F), procyanidin B3 and theobromine have strong correlations with bitterness and astringent tastes [3]. In addition, different production areas, different tea cultivars and tenderness levels of fresh leaves influence the different qualities of the taste and aroma of white tea [4,5]. Therefore, it is significant and valuable to study the taste and quality characteristics of white teas of different varieties. The sour, bitter, umami, salty and sweet taste of Fuding white tea are the highest among three production regions, while the astringency value of Yunnan white tea is significantly higher than that of Fuding and Xinyang white tea [4]. In different grades of white tea, the total contents of catechins, hydrolyzed tannins, phenolic acids, theanine and caffeine decrease with reducing grades, and theaflavins and flavonols in longevity plum and White Peony are higher than other grades [5,6]. 

Baiyun 0492 (also known as Baiyuntezao), a new cultivar of tea plant selected by individual plant selection in the Tea Research Institute of Fujian Academy of Agricultural Sciences in Wuqu Town, Shouning County, Fujian Province, has the characteristics of early spring shoot germination, and it is suitable for making white tea and green tea. The white tea manufactured from this plant exhibits superior quality compared to that from Fuding Dabai tea, suggesting its potential for being selected and bred into a distinctive elite cultivar of white tea. This study aims to analyze the differences in non-volatile substances between Baiyun 0492 and Fuyun No. 6 from the perspectives of sensory evaluation scores and physicochemical component contents based on widely-targeted metabolomics. It also explores the differential metabolites, their metabolic pathways, and the relationships between metabolites and the umami and bitterness of white tea. By doing so, it analyzes the main reasons that may cause the differences in the taste quality of white tea, with the expectation of clarifying the taste quality characteristics of Baiyun 0492 white tea and providing references for the research and development of Baiyun 0492 characteristic white tea products.

## 2. Materials and Methods

### 2.1. Tea Samples and Chemicals

In March 2022, fresh tea leaves of Baiyun 0492 (BY) and Fuyun 6 (FY) cultivars were carefully harvested from an experimental base situated in Ningde City, Fujian Province, China. Depending on the specific picking criteria—single bud, one bud accompanied by one leaf, and one bud accompanied by two leaves—these freshly collected tea leaves were meticulously processed into three distinct types of white tea: White Tip Silver, special-grade White Peony, and first-grade White Peony. Subsequently, the processed tea samples were named BY01, BY02, BY03, FY01, FY02, and FY03, sequentially indicating their quality ranking from the highest to the lowest grade.

The production process of the white tea adhered to a standardized two-stage procedure. Initially, the fresh tea leaves underwent withering at a precisely controlled temperature of 25 °C for a duration of 48 h. This was followed by a drying process, during which the withered leaves were heated at 80 °C for 20–30 min. The drying step was carefully monitored to ensure that the final moisture content of the tea products was reduced to around 5%, which is an optimal level for preserving the unique flavor and quality characteristics of white tea. After removing impurities, the samples were sealed and stored for future use. Three independent biological replicates were established to ensure the reliability and reproducibility of the experimental results.

All the following reagents used in this study had an analytical purity greater than 98%. Methanol and acetonitrile were procured from Merck & Co., Inc. (Kenneworth, NJ, USA), while formic acid was obtained from Aladdin Bio-Chem Technology Co., Ltd. (Shanghai, China).

### 2.2. Sensory Evaluation and Quantitative Descriptive Analysis (QDA)

The tea samples were evaluated and scored by eight professional sensory assessment panelists from the Tea Research Institute of Fujian Academy of Agricultural Sciences. These panelists, consisting of four females and four males aged between 30 and 50 years old, had over five years of experience in descriptive sensory analysis of tea.

The evaluation was carried out in strict accordance with the Methodology for the Sensory Evaluation of Tea (GB/T 23,776-2018; [7]). Specifically, six white tea samples, each weighing 3 g, were precisely measured and placed into 150-mL cylindrical cups. Subsequently, these samples were brewed with boiling water for 5 min. After brewing, the tea infusions were carefully filtered and then subjected to evaluation by the panel.

Quantitative descriptive analysis (QDA) was performed following the procedures outlined in previous studies [8]. The brewed tea infusions were randomly assigned three-digit codes and presented to the eight evaluators in a random order. The evaluators scored the samples on a scale ranging from 0 to 10, assessing various taste descriptors including sweetness, mellowness, bitterness, umami, astringency, sourness, aftertaste sweetness, aftertaste, thickness, and off-flavor (Table 1). The taste intensity was categorized as follows: 0–2 indicated very weak, 2–4 indicated weak, 4–6 indicated moderate, 6–8 indicated strong, and 8–10 indicated very strong. Each expert panelist evaluated each sample three times at separate time points to ensure the reliability and consistency of the assessment results.

### 2.3. Sample Preparation and Extraction

Biological samples were subjected to freeze-drying using a vacuum freeze-dryer (Scientz-100F, Scientz, Ningbo, China). Subsequently, the freeze-dried samples were pulverized in a mixer mill (MM 400, Retsch, Haan, Germany) with the aid of a zirconia bead. The milling process was carried out at a frequency of 30 Hz for 1.5 min.

Fifty milligrams of the obtained lyophilized powder was then dissolved in 1.2 mL of a 70% methanol solution. The mixture was vortexed for 30 s at 30-min intervals, with a total of six vortexing operations.

After that, the solution was centrifuged at 12,000× *g* revolutions per minute (rpm) for 3 min. The resulting extracts were filtered through a membrane filter (SCAA-104, with a pore size of 0.22 μm, sourced from ANPEL, located in Shanghai, China; website: http://www.anpel.com.cn/) prior to undergoing ultra-performance liquid chromatography-tandem mass spectrometry (UPLC-MS/MS) analysis.

### 2.4. UPLC and ESL-Q TRAPMS/MS Conditions

The sample extracts were analyzed via a UPLC–ESI–MS/MS system (UPLC: ExionLCrM AD, available at https://sciex.com.cn/; MS: Applied Biosystems 65,000 TRAP, accessible at https://sciex.com.cn/). The detailed analytical conditions were as follows:

UPLC Conditions:

- Column: An Agilent SB-C18 column with a particle size of 1.8 µm, an inner diameter of 2.1 mm, and a length of 100 mm was utilized.
- Mobile Phase: Solvent A was pure water containing 0.1% formic acid, while solvent B was acetonitrile with 0.1% formic acid.
- Gradient Program: The analysis started with an initial mobile-phase composition of 95% A and 5% B. Over a period of 9 min, a linear gradient was applied to reach a composition of 5% A and 95% B. This 5% A and 95% B composition was maintained for 1 min. Subsequently, within 1.1 min, the composition was adjusted back to 95% A and 5.0% B, which was then held for 2.9 min.
- Flow Rate: The flow velocity of the mobile phase was set at 0.35 mL per minute.
- Column Temperature: The column oven was maintained at 40 °C.
- Injection Volume: A volume of 2 μL of the sample was injected into the system.

The effluent from the UPLC was alternately connected to an ESI-triple quadrupole-linear ion trap (OTRAP)–MS for mass spectrometric analysis.

The operating parameters of the ESI source were configured as follows: The source temperature was maintained at 500 °C. In the positive ion mode, the ion spray voltage (IS) was set to 5500 V, while in the negative ion mode, it was −4500 V. The ion sources gas I (GSI), gas II (GSII), and curtain gas (CUR) were adjusted to 50 psi, 60 psi, and 25 psi, respectively. The collision-activated dissociation (CAD) was set to a high level.

Q1Q3 scans were obtained through multiple reaction monitoring (MRM) experiments. During these experiments, the collision gas (nitrogen) was set to a medium level. For each individual MRM transition, the declustering potential (DP) and collision energy (CE) were determined through further optimization of these parameters.

Based on the metabolites eluting within each specific time period, a distinct set of MRM transitions was carefully monitored. This allowed for the targeted detection and quantification of the relevant metabolites at different stages of the chromatographic separation.

### 2.5. Statistical Analysis

The mass spectrometry data were processed with Analyst 1.6.3 software. Metware spectrometry, relying on the Meiwei metabolic database, was employed to conduct qualitative and quantitative analyses of the metabolites in the samples. MultiQuant software was utilized to open the sample mass spectrometry files, integrate chromatographic peaks, and perform peak correction. To guarantee qualitative and quantitative accuracy, the mass spectrometry peaks of each metabolite detected in different samples were calibrated. All experiments were carried out with at least three biological replicates.

Heatmaps and hierarchical cluster analysis (HCA) were generated using TBtools (https://github.com/CJ-Chen/TBtools, accessed on 10 June 2025). Principal component analysis (PCA) and orthogonal partial least squares discriminant analysis (OPLS-DA) were executed via SIMCA 14.1 software (Umetrics, Umeå, Sweden), and the variable importance in projection (VIP) scores for predictor variables were calculated. Analysis of variance and significance difference analysis were performed using SPSS 26.0 (IBM, Chicago, IL, USA). In the figures, different letters denote significant differences, determined by VIP ≥ 1 and *p* < 0.05.

Pearson correlation analysis was applied to explore the relationship between differentially abundant metabolites and the quantitative values of flavor descriptions, yielding a correlation coefficient ranging from -1 to 1. In correlation analysis, the *p*-value indicates the statistical significance of the sample data. Generally, *p* < 0.05 is considered significant, and *p* < 0.01 is highly significant.

## 3. Results and Discussion

### 3.1. Sensory Evaluation Results of White Tea

**Figure 1:**
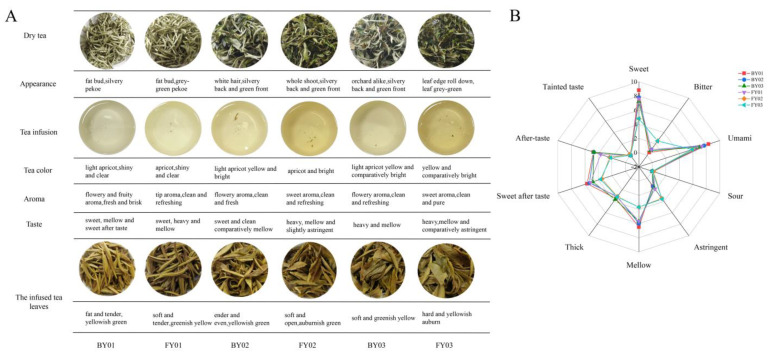
Traditional sensory evaluation for BY01, BY02, BY03, FY01, FY02, FY03 white tea (**A**). Radar plot of the characteristic aroma profile of all white tea samples (**B**).

The sensory evaluation of different white tea samples was meticulously conducted, and the results are presented in Figure 1A. Analytical findings revealed that the gustatory characteristics of Baiyun 0492 white tea were predominantly marked by sweetness and mellowness. In contrast, the taste profile of Fuyun 6 white tea was primarily characterized by a rich and full-bodied mellowness. Specifically, both BY01 and FY01 samples exhibited sweetness. However, a distinct difference was observed in their aftertaste and overall flavor balance. BY01 was notable for its pronounced sweet aftertaste, which lingered on the palate, while FY01 demonstrated a more intense and rounded mellowness, contributing to a more complex and harmonious flavor experience. When comparing BY02 and FY02, significant differences in taste were also identified. BY02 presented a sweet and clean-tasting profile, with a pure and unadulterated flavor. Conversely, FY02 had a mellow flavor profile with a subtle yet discernible astringency, adding a unique dimension to its taste. Regarding the olfactory attributes, the aroma of Baiyun 0492 white tea was predominantly floral, accompanied by delicate fruity undertones, creating a pleasant and refreshing olfactory experience. In contrast, Fuyun 6 white tea was characterized by a sweet and clear aroma, which was both inviting and distinct, contributing to its overall sensory appeal.

As the grades of BY and FY white teas decreased, their astringency and bitterness scores increased (Figure 1B). The tea infusions of BY01 were relatively sweet and mellow. However, BY02 and BY03 exhibited relatively stronger astringency, with a more intense and less pleasant taste profile. For the FY series, FY01 tea infusions had relatively high scores in terms of sweetness and sweet aftertaste, suggesting a more palatable and satisfying flavor. In contrast, FY02 and FY03 had elevated astringency and bitterness scores, which might be less preferred by some consumers.

As depicted in Figure 1A, as the grade of the two white tea varieties decreased, the color of their teas deepened, while the clarity and brightness declined. Generally, sweet, mellow, and floral aromas are the key quality attributes of BY white tea. On the other hand, mellow and sweet aromas are characteristic of FY white tea. The dry tea of BY appears silver–white, presenting an elegant and distinct visual appearance. In comparison, FY white tea is a dark green color.

The characteristic of mellowness was evident in the taste of white tea infusions, indicating that it is a common attribute among white tea infusion tastes. However, different white tea varieties vary in terms of umami, sweetness, mellowness and thickness. For instance, the infusion color of BY 0492 white tea is light yellow, while that of F6 white tea is yellow, and the latter’s clarity and brightness are relatively inferior.

### 3.2. Widely Targeted Metabolomics Analysis of Baiyun 0492 and Fuyun 6 White Tea

In order to explore the taste disparities among different white tea varieties, a comprehensive ultra-high performance liquid chromatography-tandem mass spectrometry (UPLC-MS/MS) untargeted metabolic profiling analysis was conducted on three grades of Baiyun 0492 and Fuyun 6 white tea samples. As a result, a total of 1083 metabolites belonging to 12 distinct classes were successfully identified (Table 2). Among these metabolites, a significant number are potentially associated with the taste characteristics of tea, encompassing flavonoids, amino acids and their derivatives, nucleotides and their derivatives, alkaloids, organic acids and other primary and secondary metabolites.

**Figure 2:**
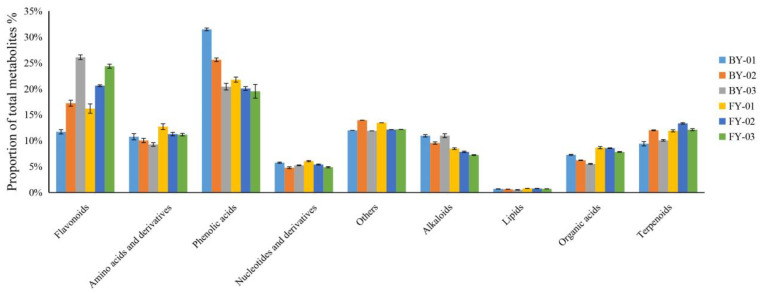
Proportional analysis of non-volatile metabolites in Baiyun 0492 and Fuyun 6 white teas.

As illustrated in Figure 2, in Baiyun 0492 (BY) and Fuyun 6 (FY) white tea samples, the proportion of flavonoid metabolites relative to the total metabolite pool exhibited an increasing trend as the white tea grade decreased. Conversely, the proportions of phenolic acid, amino acid, and organic acid metabolites declined with the decrease in white tea grade. Regarding nucleotide and alkaloid metabolites, their proportions decreased consistently with the reduction in white tea grade in FY samples. However, in BY samples, these proportions initially decreased and subsequently increased. In BY and FY samples, the proportion of lipid metabolites reached its peak in the special-grade White Peony white tea, while minimal differences were observed between the other two grades.

In BY and FY white tea samples, no statistically significant differences were observed in the proportion of terpene metabolites. Notably, the proportions of flavonoids, amino acids, and their derivative metabolites were relatively lower in Baiyun 0492, whereas Fuyun 6 exhibited a higher proportion of flavonoid metabolites. Flavonoids are recognized as crucial determinants of tea bitterness [9]. Specifically, flavonol monosides serve as key components of tea astringent compounds [10]. These findings suggest that disparities in primary metabolites among different white tea varieties may underlie the distinct taste profiles, with Baiyun 0492 white tea characterized by a sweet flavor and Fuyun 6 by a mellow taste.

As presented in Table 2, the distinctive metabolite 3,4,5-tricaffeoylquinic acid was exclusively detected in F6 white tea. This phenolic acid, formed by the condensation of caffeic acid and quinic acid, is a bioactive component present in various Chinese medicinal materials, vegetables and fruits [11]. Moreover, 3,4,5-tricaffeoylquinic acid holds promise in research on to age-related diseases. During tea processing, caffeoyl quinic acid undergoes thermal decomposition into caffeic acid and quinic acid, and quinic acid is known to contribute to the bitterness of coffee [12,13]. This may account for the more pronounced mellow and bitter taste of F6 white tea.

Conversely, N-feruloyl tyramine, a unique metabolite, was identified in BY white tea. Feruloyl tyramine demonstrates a broad spectrum of pharmacological activities, including inhibition of platelet aggregation, antioxidant properties through free radical scavenging, suppression of tumor metastasis, and notable inhibitory effects on human Hep G2 cells [14,15].

### 3.3. PCA and OPLS-DA Between Baiyun 0492 White Tea and Fuyun 6 White Tea

**Figure 3:**
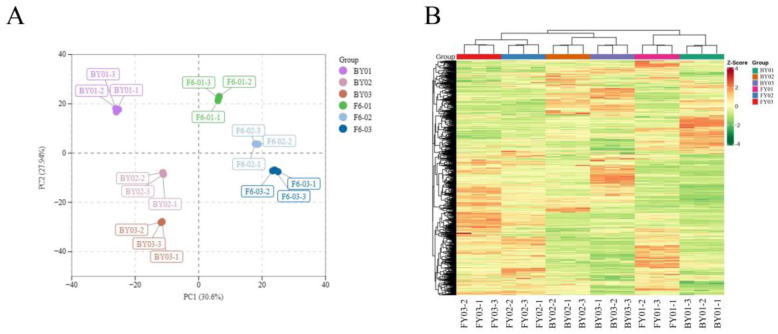
PCA score plot (**A**) and heatmap (**B**) of all white tea samples.

The principal component analysis (PCA) results of the samples are presented in Figure 3. As shown in Figure 3A, Baiyun 0492 white tea and Fuyun 6 white tea exhibited distinct separation patterns, not only between different varieties but also among different grades within the same variety. To gain a more detailed understanding of the metabolite variations in Baiyun 0492 and Fuyun 6 white tea, the peak areas of individual metabolites were normalized using ultraviolet (UV) absorbance, followed by hierarchical cluster analysis (Figure 3B). Both principal component analysis and cluster analysis effectively illustrated the overall differences in metabolite profiles between Baiyun 0492 and Fuyun 6 white tea.

Principal component analysis (PCA) was carried out on samples BY01, BY02, BY03, FY01, FY02, and FY03 to comprehensively assess the overall metabolic disparities among the samples of each group and the variability within the samples of the same group. As depicted in Figure 3A, the contribution rates of the first principal component (PC1) and the second principal component (PC2) were 30.6% and 27.94%, respectively. The clear separation trend of metabolites between the two varieties of white tea, namely Baiyun 0492 (BY) and Fuyun 6 (FY), strongly suggests significant differences in their metabolic levels.

Furthermore, the concentrated distribution of the repeated samples within each group indicates a high degree of correlation and excellent reproducibility among the samples in the same group. This validates their suitability for subsequent analysis of differentially abundant metabolites, ensuring the reliability and accuracy of the results obtained from the subsequent studies.

To conduct a more in-depth analysis of the differentially abundant metabolites between the two white tea varieties, orthogonal partial least squares discriminant analysis (OPLS-DA) models were established for three groups of white tea samples that shared the same grade but belonged to different varieties. The content data of a total of 1084 metabolites were then subjected to examination using these models. Through the application of this analytical approach, a comprehensive analysis of the 1084 metabolite content data was carried out.

Table 3 shows the evaluation parameters of the OPLS-DA. Significantly, both the R2y (cumulative explained variance of the response variable) and Q2 (cumulative predictive ability) values of the models exceeded 0.9. This outcome strongly indicates that the three sets of models were well-constructed, possessing high explanatory power for the data. Moreover, it implies that the prediction of samples based on these models is highly reliable, providing a solid foundation for the identification and interpretation of the differentially abundant metabolites between the two white tea varieties.

### 3.4. Analysis of Different Metabolites of Different Varieties of White Tea

#### 3.4.1. Screening of Differentially Abundant Metabolites

**Figure 4:**
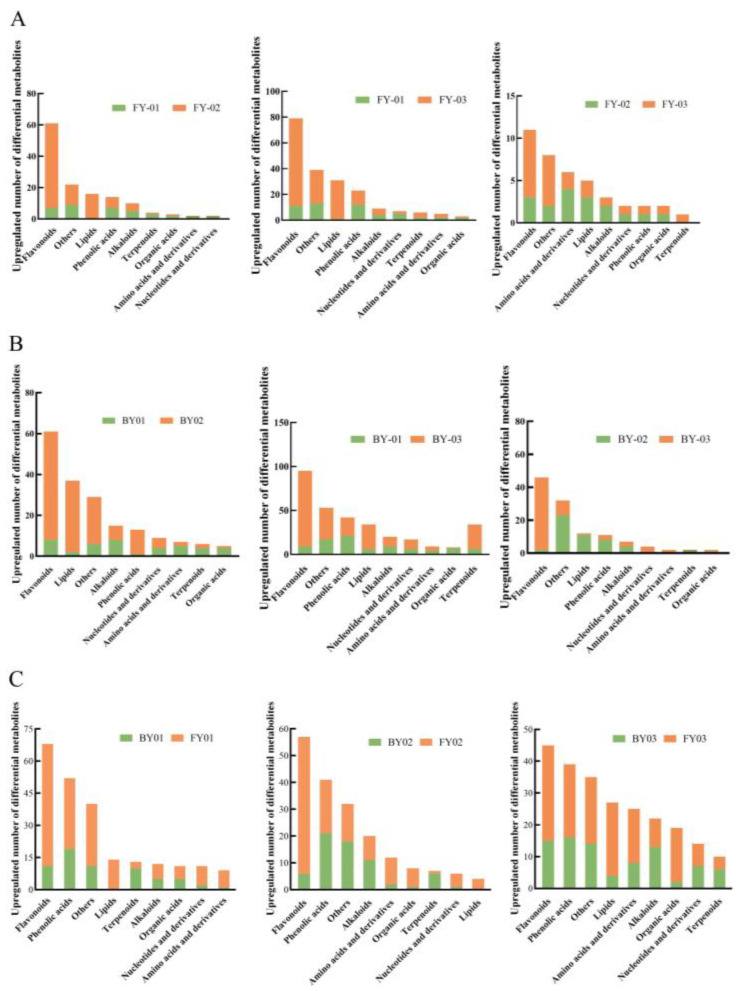
Different metabolites of Baiyun0492 and Fuyun 6 white tea in the same grade (**A**), Baiyun0492 white tea in different grades (**B**), Fuyun 6 white tea in different grades (**C**).

To systematically analyze the characteristics of differentially abundant metabolites among different white tea varieties, an orthogonal partial least squares discriminant analysis (OPLS-DA) model was employed with the criteria of variable importance in the projection (VIP) ≥ 1 and a *p*-value < 0.05. In Fuyun 6 white tea, 134, 202 and 40 differentially abundant metabolites were identified across the three grades, respectively. These differentially abundant metabolites accounted for 12.37%, 18.65% and 3.69% of the total 1083 metabolic components in the three grades of Fuyun 6 white tea, as illustrated in Figure 4. The predominant classes of differentially abundant metabolites in Fuyun 6 were flavonoids, other compound classes and lipids.

For Baiyun 0492 white tea, 188, 283 and 118 differentially abundant metabolites were detected in the corresponding three grades. The proportions of these differentially abundant metabolites relative to the total 1083 metabolic components were 17.36%, 26.13% and 10.90%, respectively. The major categories of differentially abundant metabolites in Baiyun 0492 white tea were flavonoids, lipids and phenolic acids.

When comparing the three grades of two white tea varieties within the same species, the number of upregulated differentially abundant metabolites in White Tip Silver was consistently lower than that in both the special-grade and first-grade White Peony, with the most significant difference observed between White Tip Silver and first-grade White Peony. Specifically, in Fuyun 6 white tea, 150 upregulated differentially abundant metabolites were identified in the White Tip Silver (FY03) grade, accounting for 74.26% of the total differentially abundant metabolites in the special-grade (FY01) White Peony. Similarly, in Baiyun 0492 white tea, 201 upregulated metabolites were detected in the White Tip Silver (BY03) grade, constituting 71.02% of the total differentially abundant metabolites in the special-grade (BY01) White Peony.

Although the differences between the special-grade and first-grade White Peony were relatively minor, the first-grade White Peony exhibited a higher number of upregulated differentially abundant metabolites. Notably, most of the differentially abundant metabolites showed a negative correlation with the grades of Baiyun 0492 and Fuyun 6 white tea. The relative content of these metabolites was the lowest in the White Tip Silver samples and increased as the grade decreased. This finding aligns with previous research [16], which demonstrated that the total flavonoid glycoside content of white tea increases with the increasing maturity of the tea leaves.

Analysis revealed 230, 187 and 236 distinct metabolites in the same-grade white tea of the two varieties, predominantly flavonoids, phenolic acids and amino acids. These metabolites accounted for 21.24%, 17.27% and 21.79% of the total metabolic components, respectively, confirming significant metabolic disparities between the two varieties at each grade. Flavonoid metabolites emerged as key discriminators for differentiating the two white tea varieties.

Compared with Baiyun 0492, the majority of differentially abundant metabolites in Fuyun 6 white tea were upregulated. Specifically, 166 metabolites in Fuyun 6 White Tip Silver, 121 in special-grade White Peony and 151 in first-grade White Peony were upregulated, representing 72.17%, 64.71% and 63.98% of the total differentially abundant metabolites between the two varieties, respectively. These grade-specific differences in metabolite types and contents contributed to the distinct taste characteristics of the two varieties [17,18].

Seasonal variations likely influenced these metabolic differences. As reported by Yu et al., the contents of tea polyphenols (TP), caffeine (TC), epicatechin gallate (ECG) and epigallocatechin gallate (EGCG) increase during spring, and elevated temperature and light intensity promote the accumulation of specific flavonoids and their glycosides [19]. Quercetin and its glycosides also increase from early to late spring [20]. Given that Baiyun 0492, an early-maturing variety, is harvested earlier than Fuyun 6, this temporal difference in harvesting may partially explain the metabolic disparities between the two varieties.

#### 3.4.2. Functional Annotation and Enrichment Analysis of Differentially Abundant KEGG Metabolites in Various White Tea Cultivars

To further understanding of the metabolic pathway-level differences in metabolites among different grades of the two white tea varieties, pathway attribution analysis was conducted to map the metabolic pathways corresponding to the differentially abundant metabolites. These metabolites interact in vivo, forming distinct pathways, and group-specific metabolic pathway differences were thereby identified.

The Kyoto Encyclopedia of Genes and Genomes (KEGG) database was employed to annotate the differentially abundant metabolites, revealing that they were predominantly involved in primary metabolic pathways and the biosynthesis of secondary metabolites. Additionally, KEGG-based pathway enrichment analysis was performed on these differentially abundant metabolites. A total of 230, 187 and 236 significantly different metabolites, identified in the White Tip Silver, special-grade White Peony and first-grade White Peony white tea of the two varieties, respectively, were mainly distributed across 20 metabolic pathways (Figure 4).

A total of 68 significantly differential metabolites were screened from the two white tea varieties and annotated to 64 metabolic pathways using the Kyoto Encyclopedia of Genes and Genomes (KEGG) database. The top five most enriched pathways were flavonoid biosynthesis, starch and sucrose metabolism, carbon fixation in photosynthetic organisms, glycolysis and cofactor biosynthesis. Flavonoid biosynthesis, a pathway closely associated with white tea quality, involved 15 distinct metabolites, such as kaempferol, quercetin, naringenin-7-O-neohesperidoside (naringin), apigenin-8-C-glucoside (vitexin) and eriodictyol. In FY01, the levels of these metabolites were significantly upregulated by 1.36-fold, 1.3-fold, 4.67-fold, 1.33-fold and 2.07-fold, respectively. Naringin, the primary compound responsible for the bitterness of citrus fruits, still imparts bitterness at a concentration of 20 mg/kg in aqueous solution [21,22]. Flavonols predominantly exist in tea as glycosides of kaempferol and quercetin, accounting for 2–3% of the tea’s weight [23,24].

For the special-grade White Peony white tea of the two varieties, 52 significantly differential metabolites were identified, and KEGG analysis assigned them to 51 metabolic pathways. The top five pathways with the highest abundance of differentially expressed metabolites were flavonoid biosynthesis, stilbenoid, diarylheptanoid and gingerol biosynthesis, galactose metabolism, caffeine metabolism, and phenylpropyl biosynthesis. Among them, flavonoid biosynthesis, galactose metabolism, and caffeine metabolism were related to white tea quality, involving 19 metabolites. In FY02, the levels of quercetin, naringenin-7-O-neohesperidin (naringenin) and luteolin were significantly upregulated by 1.62-fold, 2.57-fold and 3.11-fold, respectively. Luteolin and apigenin are major components of tea flavonoids [25].

In BY02, lactobiose, meliobiose, raffinose, D-sucrose, 1,7-dimethylxanthine and theophylline were significantly upregulated by 1.42-fold, 1.53-fold, 1.51-fold, 1.50-fold, 1.50-fold and 1.88-fold, respectively. Carbohydrates, encompassing monosaccharides, disaccharides, polysaccharides and their derivatives, constitute a substantial portion of white tea (20–25% of dry weight) and are regarded as the basis for its sweetness and aroma [26]. Deng et al. reported that the trend of total soluble sugar content was inversely correlated with that of flavonoids, possibly due to the frequent binding of soluble sugars to flavonoids in the form of glycosides [27]. Theophylline, a positive inotropic agent, exhibits therapeutic efficacy for various respiratory diseases and is a common medication for asthma. Notably, white tea contains higher levels of theophylline compared to green and yellow teas [28]. 1,7-Dimethylxanthine, a natural dietary component, serves as the primary metabolite of caffeine in the human body [29].

From the first-grade White Peony of the two cultivars, 77 significantly differential metabolites were selected. KEGG analysis revealed that the top five most enriched pathways were flavonoid biosynthesis, alpha-linolenic acid metabolism, amino acid metabolism, caffeine biosynthesis and purine metabolism. Metabolic pathways related to white tea quality, namely flavonoid biosynthesis, amino acid metabolism and caffeine metabolism, involved 23 metabolites. In FY03, the levels of quercetin, naringenin-7-O-neohesperidin (naringenin), eriodictyol, L-asparagine, L-ornithine and L-arginine were significantly upregulated by 1.53-fold, 1.90-fold, 1.35-fold, 1.31-fold and 3.39-fold, respectively. Although asparagine is present at relatively high concentrations in white tea, its taste-perception threshold is extremely high [3].

### 3.5. Correlation Analysis of Differentially Abundant Metabolites and Quality of Baiyun 0492 White Tea

#### 3.5.1. Correlation Analysis of Different Metabolites and Quality of BY01 and FY01

**Figure 5:**
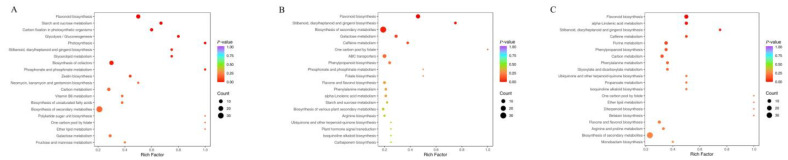
Kyoto Encyclopedia of Genes and Genomes (KEGG) enrichment map of differential metabolites. (**A**) BY01 VS FY01 (**B**) BY02 VS FY02 (**C**) BY03 VS FY03.

The correlations between the differential metabolites of White Tip Silver and tea flavor descriptors were evaluated using pairwise correlation analysis. A total of 230 differential metabolites and ten tea flavor indicators were investigated. Among them, 24 differential metabolites were found to have significant correlations with tea taste indicators. Specifically, flavonoid compounds such as quercetin-3-O-rhamnoside (quercitrin), apigenin-6-C-arabinoside-8-C-xyloside, apigenin-6,8-di-C-arabinoside and myricetin-3-O-(6”-malony) glucoside exhibited a significant negative correlation with umami taste (Figure 5). Flavonoids, predominantly existing as glycosides of kaempferol, quercetin and myricetin in tea, not only significantly impact the astringent and velvety texture of tea but also contribute to its bitterness [30]. The higher relative content of these substances in FY01 compared to BY01 may partially account for the bitterness of FY01.

Among the amino acids, L-Alanyl-L-Alanine showed a significant positive correlation with sweetness. Amino acids and their derivative compounds are intricately associated with tea aroma, as well as its sweet, bitter, and umami tastes [31]. Different amino acids possess distinct taste characteristics; for instance, serine, alanine, threonine and glycine impart a sweet flavor [32]. In yellow tea, alanine was positively correlated with aftertaste and sweetness [33]. Additionally, diosmin and 3,4,2’,4’,6’-pentahydroxychalcone-4’-O-glucoside were significantly positively correlated with sweetness. Diosmin exhibits a wide range of pharmacological activities, including anti-inflammatory, antioxidant, antidiabetic, anticancer, antimicrobial, hepatoprotective, neuroprotective, cardioprotective, renoprotective and retinoprotective effects [34]. The higher relative content of these substances in BY01 compared to FY01 may contribute to the greater sweetness of BY01 relative to FY01.

#### 3.5.2. Correlation Analysis of Different Metabolites and Quality of BY02 and FY02

The correlations between the differentially abundant metabolites of special-grade White Peony and tea taste descriptors were evaluated through pairwise correlation analysis. A total of 187 differentially abundant metabolites and ten tea taste indices were investigated. Notably, 183 of these metabolites showed significant correlations with the tea taste indices.

Among the amino acids, L-arginine, homoproline, and N-α-acetyl-L-ornithine exhibited significant negative correlations with sweetness, umami, and mellowness. Specifically, L-arginine was significantly positively correlated with bitterness, as it is known to contribute to the bitter taste in tea [35]. Regarding phenolic acids, isochlorogenic acid A, isochlorogenic acid B and isochlorogenic acid C were significantly positively correlated with the sour taste. Conversely, 3,4,5-tricaffeoylquinic acid, neochlorogenic acid (5-O-caffeoylquinic acid) and phenethyl caffeate showed significant negative correlations with the sweet and fresh tastes. Isochlorogenic acids A, B and C are the major polyphenolic compounds in Kuding tea, which is characterized by its bitter flavor [36]. Among flavonoids, compounds such as pinocembrin, quercetin, catechin, naringenin-7-O-neohesperidoside (naringin), spiraeoside and quercetin-3-O-rhamnoside (quercitrin) had significant negative correlations with sweetness. Catechins, flavonoids and flavonols are key contributors to the bitterness and astringency of tea, and they are prone to degradation, oxidation and hydrolysis under high-temperature conditions [37]. Naringin, a common plant flavonoid glycoside, also imparts a bitter taste to tea [38]. Among organic acids, jasmonic acid was significantly negatively correlated with freshness, umami, mellowness and sweet aftertaste. Organic acids serve as important intermediate components in carbohydrate catabolism and significantly influence the taste and aroma of tea [39]. Given that the relative content of these substances was higher in FY02 than in BY02, this may explain why FY02 exhibits a more pronounced bitterness compared to BY02. On the other hand, certain phenolic acids, including chlorogenic acid (3-O-caffeoylquinic acid) and cryptochlorogenic acid (4-O-caffeoylquinic acid), were significantly positively correlated with sweetness and umami. A reduction in phenolic acid content is beneficial for transforming the tea taste from astringent to mellow [40]. Currently, it is well-documented that polyphenols are the primary astringent substances in tea, with ellagic acid and chlorogenic acid being the main phenolic acids [41]. Additionally, meliose, D-maltose and D-sucrose were positively correlated with sweetness. Soluble sugars, as the main water-soluble carbohydrates in tea, play a crucial role in determining its sweetness, with sucrose, fructose and glucose being the predominant sugars [42]. Since the relative content of these sugars was higher in BY02 than in FY02, this could account for the greater sweetness of BY02 compared to FY02.

#### 3.5.3. Correlation Analysis of Different Metabolites and Quality of BY03 and FY03

The correlations between the differentially abundant metabolites of first-grade White Peony and tea taste descriptors were evaluated via pairwise correlation analysis. A total of 236 differentially abundant metabolites and ten tea taste indices were examined. Notably, 230 of these metabolites showed significant correlations with the tea taste indices (Table 1).

Among the amino acids, L-arginine, homoproline, N-α-acetyl-L-ornithine, L-methionine-L-serine (Met-Ser), and L-asparagine exhibited significant negative correlations with sweetness. Synthetic asparagine analogs are known to have a distinctly bitter taste [43]. Regarding phenolic acids, isochlorogenic acid A, isochlorogenic acid B, and isochlorogenic acid C were significantly positively correlated with musty and sour tastes. Conversely, 3,4,5-tricaffeoylquinic acid, phenethyl caffeate and neochlorogenic acid (5-O-caffeoylquinic acid) were significantly negatively correlated with sweetness. Phenolic acids are crucial for tea’s health-promoting properties, essential for the synthesis of catechins and flavonols, and contribute to the bitter and astringent tastes of tea [3,44,45]. They also play a vital role in determining tea’s taste and color [44]. Among flavonoids, compounds such as monasin, quercetin, catechin, naringin, spiraeoside, quercetin-7-O-glucoside and eriocitrin showed significant negative correlations with sweetness. Tea flavonols, mainly including quercetin, kaempferol and myricetin, influence both the astringency and color of tea. Catechins are the primary contributors to the astringency and bitterness of brewed tea [46,47]. Given that the relative content of these substances was higher in FY03 than in BY03, this may partly explain the more bitter taste of FY03 compared to BY03. On the other hand, certain amino acids, including L-tyrosine, n-acetyl-L-tyrosine, n-acetyl-L-phenylalanine, L-cyclopentylglycine, o-acetylserine, S-methyl-l-cysteine and 3-hydroxy-l-phenylalanine, were significantly positively correlated with sweet aftertaste. While the tyrosine monomer solution itself has no flavor, its relationship with tea flavor remains unclear [33]. Among phenolic acids, chlorogenic acid and cryptochlorogenic acid showed significant positive correlations with sweetness. Cryptochlorogenic acid and isochlorogenic acid A are isomers of chlorogenic acid. In nucleotides, hypoxanthine and 1,7-dimethylxanthine were positively correlated with sweetness. Hypoxanthine, primarily formed through the metabolism of adenine and guanine and a key precursor in tea caffeine synthesis [48], can be bioconverted to theophylline and 1,7-dimethylxanthine in oolong tea [49]. In other metabolite categories, meliose and D-maltose were significantly positively correlated with sweetness. An increase in soluble sugar content enhances the sweetness of the tea, reduces its bitterness and astringency, and increases its viscosity [33], which likely contributes to the sweet and mellow taste of BY03 tea.

#### 3.5.4. Correlation Analysis of Shared Metabolites and Quality Traits Between Baiyun 0492 and Fuyun 6

**Figure 6:**
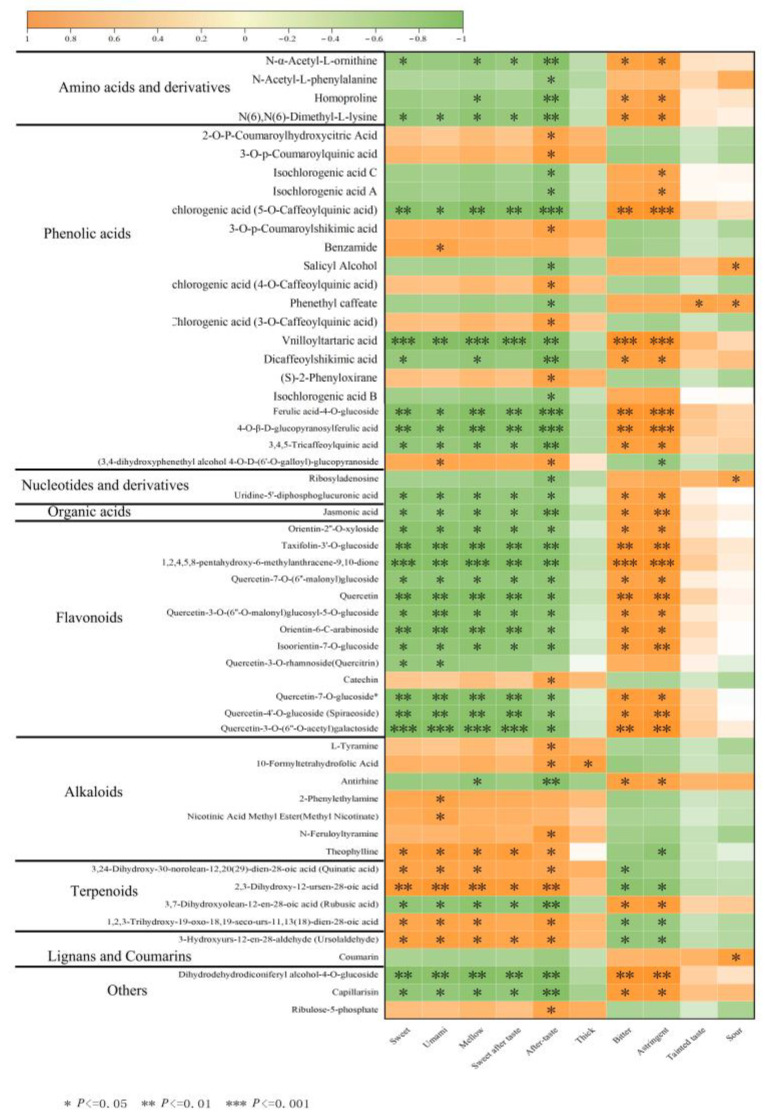
Correlation analysis of different metabolites and quality indexes in white tea.

A correlation analysis was performed between the common differentially abundant metabolites identified across three samples of Baiyun 0492 and Fuyun 6 white tea at different grades and the quantitative taste descriptors. Integrating the findings, a total of 55 common differentially abundant metabolites were determined to exhibit significant correlations with the quantitative taste descriptors (*p* < 0.05, VIP > 1) (Figure 6).

The majority of flavonoid-related differences showed negative correlations with sweetness, freshness, mellowness, sweet aftertaste and overall aftertaste, while demonstrating positive correlations with bitterness and astringency. Flavanols, which are ubiquitously present in tea, are known to contribute to its bitter and astringent characteristics [50]. Among phenolic acids, 3,4,5-tricaffeoylquinic acid and neochlorogenic acid (5-O-caffeoylquinic acid) were negatively correlated with sweetness, astringency, mellowness and aftertaste, but positively correlated with bitterness and astringency. Isochlorogenic acid A and isochlorogenic acid C were positively correlated with astringency. As non-flavonoid polyphenols in tea, phenolic acids contribute to the acidity and umami taste of brewed tea [3,51]. Neochlorogenic acid, an ester formed by the reaction between the 5-hydroxyl group of caffeic acid and L-quininic acid, plays a role in these taste correlations. Regarding amino acids, L-arginine was negatively correlated with sweetness, mellowness and aftertaste, and positively correlated with bitterness and astringency. Catechins showed a significant positive correlation with aftertaste. Among catechins, ester catechins are associated with a strong bitter sensation and intense astringency, serving as the main contributors to astringent perception, whereas non-ester catechins have a milder astringency, weaker astringent effects, and contribute to a pleasant aftertaste, potentially due to their higher content [52,53]. These metabolic differences may account for the sweeter taste profile of Baiyun 0492 compared to Fuyun 6.

## 4. Conclusions

This study employed UPLC-MS/MS to compare nonvolatile metabolites in Baiyun 0492 and Fuyun 6 white teas. Significant inter-varietal differences in nonvolatile metabolite profiles were observed, with bioactive *N*-feruloyl tyramine identified as a potential characteristic metabolite of Baiyun 0492, and bitter 3,4,5-tricaffeoylquinic acid as a marker for Fuyun 6. Most differentially abundant metabolites negatively correlated with tea grade, showing lowest levels in White Tip Silver samples and increasing with decreasing grade. Flavonoids, phenolic acids and amino acids differed significantly between varieties at the same grade. KEGG pathway analysis highlighted enrichment in flavonoid, caffeine, amino acid and carbohydrate metabolism.

Widely-targeted metabolomics combined with taste descriptor correlation analysis revealed most flavonoids, certain amino acids and phenolic acids (e.g., quercetin, quercitrin, L-arginine, 3,4,5-tricaffeoylquinic acid, neochlorogenic acid) positively correlated with bitterness/astringency, with higher levels in Fuyun 6. Soluble sugars (e.g., D-maltose, D-sucrose), positively linked to sweetness, were lower in Fuyun 6, explaining the sweet–mellow profile of Baiyun 0492 versus the dominant mellowness of Fuyun 6.

Comparative metabolite analysis confirmed the more pronounced sweet–fresh flavor of Baiyun 0492, providing a scientific basis for its product development. Taste characteristics were attributed to metabolite composition differences, with cultivar as the primary determinant. Future studies using targeted metabolomics and taste threshold data will further elucidate Baiyun 0492’s flavor mechanisms and the roles of key nonvolatile metabolites.

## Figures and Tables

**Table 1:** Definitions of sensory attributes for tea samples.

Attribute	Definition	Reference
Sweet	Intensity of sweet taste	Sucrose solution (0.15 mg/mL = 1; 4 mg/mL = 10)
Bitter	Intensity of bitter taste	Caffeine solution (0.05 mg/mL = 1; 0.8 mg/mL = 10)
Umami	Intensity of umami taste	Glutamic acid solution (0.04 mg/mL = 1; 0.32 mg/mL = 10)
Sour	Intensity of sour taste	Citric acid solution (0.15 mg/mL = 1; 0.8 mg/mL = 10)
Astringent	Intensity of astringent taste	Alum solution (0.1 mg/mL = 1; 0.24 mg/mL = 10)
Sweet aftertaste	After drinking tea, the root of the tongue and throat have a sweet feeling, and have a feeling of moisture.	Liquorice solution
Mellow	Shade is moderate, soft palate.	Mixed solution of pectin and sucrose
Aftertaste	The taste felt in the throat of the mouth after drinking tea	
Thick	The contents are rich and sticky.	
Tainted taste	It seems to be colloidal suspension or to contain impurities	

**Table 2:** Numbers of the main metabolites in all white tea samples.

Compounds	Amount	BY01	BY02	BY03	F6-01	F6-02	F6-03
Flavonoids	178	176 (Isoorientin-7-*O*-glucoside) (Quercetin-3-*O*-(6”-*O*-malonyl) glucosyl-5-*O*-glucoside)	176 (Isoorientin-7-*O*-glucoside) (Vitexin-7-*O*-(6”-feruloyl) glucoside-4’-*O*-glucoside)	176(Vitexin-7-*O*-(6”-feruloyl) glucoside-4’-*O*-glucoside) (Kaempferol-3-*O*-[2-*O*-(*trans*-p-coumaroyl)-3-*O*-α-D-glucopyranosyl]-α-D-glucopyranoside)	176(Luteolin-7-*O*-rutinoside) (Brassicin)	178	178
Amino acids and derivatives	119	119	119	119	119	119	119
Phenolic acids	191	189 (3,4,5-Tricaffeoylquinic acid) (3-(3-Hydroxyphenyl)-propionic acid)	189(3,4,5-Tricaffeoylquinic acid) (3-(3-Hydroxyphenyl)-propionic acid)	190 (3,4,5-Tricaffeoylquinic acid)	190 ((S)-2-Phenyloxirane)	190 (3,6-Di-*O*-caffeoyl glucose)	190 ((S)-2-Phenyloxirane)
Nucleotides and derivatives	60	60	59(5-Methyluridine)	59 (5-Methyluridine)	60	60	59(Hypoxanthine)
Quinones	14	14	14	14	14	14	14
Lignans and Coumarins	31	31	31	31	31	31	31
Others	119	117 (Vitamin K2) (*Trans*-dehydrorosinone)	119	119	117(Vitamin K2)) (*Trans*-dehydrorosinone)	118(D-Fructose)	119
Tannins	27	27	27	27	27	27	27
Alkaloids	90	90	89 (Dihydrocaffeoylputrescine)	89 (Dihydrocaffeoylputrescine)	87(Cinnamoyltyramine) (N-Feruloyltyramine) (N-(4-hydroxyphenethyl) cinnamamide)	89 (N-Feruloyltyramine)	87 (Cinnamoyltyramine)(N-Feruloyltyramine) (N-(4-hydroxyphenethyl)cinnamamide)
Terpenoids	28	27(Pimaric acid)	28	27 (Pimaric acid)	27(8-Epiloganic acid)	26 (2,3-Dihydroxy-12-ursen-28-oic acid) (Quinatic acid)	26 (2,3-Dihydroxy-12-ursen-28-oic acid) (Quinatic acid)
Organic acids	90	90	90	90	90	90	90
Lipids	136	134 (LysoPE 16:3) (10,16-Dihydroxypalmitic acid)	136	136	136	136	136
Total	1083	1074	1078	1077	1074	1077	1076

**Table 3:** Parameters of OPLS-DA analysis.

Sample	R^2^_x_	R^2^_y_	Q^2^
FY01 vs. FY02	0.616	1	0.972
FY01 vs. FY03	0.650	1	0.976
FY02 vs. FY03	0.525	1	0.939
BY01 vs. BY02	0.763	1	0.983
BY01 vs. BY03	0.718	1	0.987
BY02 vs. BY03	0.617	0.999	0.972
BY01 vs. FY01	0.683	1	0.982
BY02 vs. FY02	0.647	0.999	0.976
BY03 vs. FY03	0.692	0.999	0.984

## Data Availability

The original contributions presented in the study are included in the article. Further inquiries can be directed to the corresponding authors.
